# Amyloid β 1-42 Can Form Ion Channels as Small as Gramicidin in Model Lipid Membranes

**DOI:** 10.3390/membranes15070204

**Published:** 2025-07-08

**Authors:** Yue Xu, Irina Bukhteeva, Yurii Potsiluienko, Zoya Leonenko

**Affiliations:** 1 Department of Physics and Astronomy, University of Waterloo, Waterloo, ON N2L 3G1, Canada; 2 Waterloo Institute for Nanotechnology, University of Waterloo, Waterloo, ON N2L 3G1, Canada; 3 Department of Biology, University of Waterloo, Waterloo, ON N2L 3G1, Canada

**Keywords:** amyloid beta, gramicidin, Alzheimer’s Disease, electrophysiology, artificial lipid membrane, ion channel

## Abstract

The amyloid-beta 1-42 (Aβ1-42) oligomers are the most cytotoxic species of the amyloid family and play a key role in the pathology of Alzheimer’s Disease (AD). They have been shown to damage cellular membranes, but the exact mechanism is complex and not well understood. Multiple routes of membrane damage have been proposed, including the formation of pores and ion channels. In this work, we study the membrane damage induced by Aβ1-42 oligomers using black lipid membrane (BLM) electrophysiology and compare their action with gramicidin, known to form ion channels. Our data show that Aβ1-42 oligomers can induce a variety of damage in the lipid membranes composed of 1,2-dipalmitoyl-sn-glycero-3-phosphocholine (DPPC), 1-palmitoyl-2-oleoyl-sn-glycero-3-phosphocholine (POPC), and cholesterol (CHOL), including small ion channels, similar to the gramicidin channels, with an average inner diameter smaller than 5 Å. These channels have a short retaining time in lipid membranes, suggesting that they are highly dynamic. Our studies provide new insights into the mechanism of membrane damage caused by Aβ1-42 oligomers and extend the current perception of the Aβ channelopathy hypothesis. It provides a more in-depth understanding of the molecular mechanism by which small Aβ oligomers induce cytotoxicity by interacting with lipid membranes in AD.

## 1. Introduction

**Figure 1:**
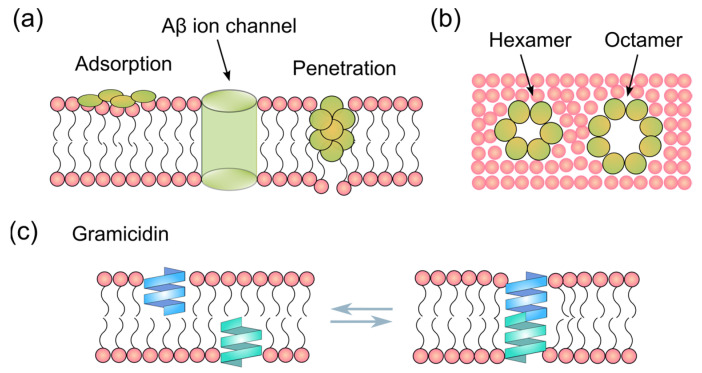
Amyloid beta (Aβ)/gramicidin –membrane interaction. (**a**) Aβ oligomers can interfere with lipid membranes via multiple mechanisms, such as adsorption, penetration, and pore or ion channel formation. (**b**) Top view of potential diverse Aβ pore formation in the lipid membrane. (**c**) Gramicidin ion channel formation with two subunits.

Soluble amyloid-beta 1-42 (Aβ1-42) oligomers have a strong cytotoxicity that contributes to neuron dysfunction and neurodegeneration in Alzheimer’s Disease [1]. They have profound membrane affinity and can disrupt the lipid membranes via several mechanisms [2,3,4,5,6,7], as indicated in Figure 1a, such as detergent-like effects extracting lipid molecules from the membrane [8], forming non-selective pores or ion channels that transport Calcium ions (Ca^2+^) and monovalent ions [9], and binding to and embedding into the lipid membranes [5,6,10]. These membrane changes caused by Aβ1-42 oligomers can lead to abnormal ionic transport, thereby disrupting Ca^2+^ homeostasis [11].

According to the channelopathy hypothesis, the formation of ion channels has been proposed as the mechanism by which Aβ damages neuronal membranes [9,12,13]. The detergent-like effect of Aβ oligomers can accelerate the degradation of supported lipid bilayers, as demonstrated by atomic force microscopy (AFM) [8] and, according to the Moir Hypothesis [14], their action may be similar to that of antimicrobial peptides [15]. Modeling studies suggest that Aβ1-42 aggregates can form β-barrel circular structures with a diameter of 1 to 2 nm [16,17], supported by EM and AFM, showing the formation of circular pores or channels [18,19]. Bode et al. experimentally recorded ion currents induced by Aβ1-42 but not Aβ1-40 in cellular membranes using a patch clamp, suggesting the formation of ion channels [20]. Their TEM and patch clamp results estimated that Aβ1-42 oligomers, with sizes ranging from 5 to 20 nm, can form ionic pores with diameters of 1.7 to 2.4 nm [20]. These experimental and simulation studies suggest that a variety of Aβ–membrane interactions can exist (Figure 1a), and the heterogeneity of Aβ aggregates (Figure 1b), whose size varies with the number of aggregated subunits [21], contributes to this complexity. CryoET studies suggested that the smallest Aβ oligomers may consist of just 3–6 Aβ molecules, appear roughly spherical in shape, and be 2–3 nm in diameter [22]. The ssNMR study conducted by Lendel et al. has described an Aβ β-barrel hexamer structure with a diameter of 3 nm [23]. Another study by Ciudad et al., combining NMR and MD simulation, suggested that pores can be formed with Aβ(1-42) tetramers and octamers [24]. Recently, Liang et al., using cryoEM and cryoET studies, reconstructed the structure of annular Aβ1-42 oligomers composed of around 12–16 Aβ molecules and confirmed their ability to form pores [19]. The work by Serra-Batiste et al. experimentally demonstrated that Aβ1-42 but not (1–40) oligomers can form ion channels with a β-barrel structure [7]. In this work, using the electrophysiology method, we provide a comparison of the Aβ1-42 ion channels with a well-characterized gramicidin D channel to estimate the size of the ionic pore.

Gramicidin D (a mixture of gramicidin A, B, and C (with gramicidin A as the majority) is a naturally occurring antimicrobial peptide produced by the bacterium Bacillus brevis [25,26]. In its natural form, two subunits of β-helical gramicidin form a dimer that can insert into lipid bilayers and form a transmembrane pore, which retains the β helicity [27], as shown in Figure 1c. This pore, with a diameter of 0.4 nm (4 Å), acts as an ion channel for monovalent cations, such as sodium and potassium ions [28]. The study of gramicidin ion channels has provided valuable insights into the mechanism of ion transport across biological membranes, contributing to an understanding of ion channel function [29]. Therefore, gramicidin, one of the most widely studied and well-defined channels, is an ideal model protein that we used in this work for comparison with ionic pores or channels formed by Aβ1-42.

In this study, we aimed to identify and classify the actions of small Aβ oligomers on the lipid membranes using atomic force microscopy (AFM) and black lipid membrane (BLM) electrophysiology. The AFM was employed to confirm the formation of small Aβ1-42 oligomers produced with Stine’s protocols. BLM was used to record the instantaneous ion current events created by Aβ1-42 oligomers and gramicidin interacting with suspended lipid membranes composed of 1,2-dipalmitoyl-sn-glycero-3-phosphocholine (DPPC), 1-palmitoyl-2-oleoyl-sn-glycero-3-phosphocholine (POPC), and cholesterol (CHOL). The Aβ –membrane interactions were classified using their ionic current signatures and compared with those of gramicidin channels. Our results show that small Aβ1-42 oligomers can form ion channels similar in size to gramicidin ion channels.

## 2. Materials and Methods

### 2.1. Reagents

Lipids, 1,2-dipalmitoyl-sn-glycero-3-phosphocholine (DPPC), 1-palmitoyl-2-oleoyl-sn-glycero-3-phosphocholine (POPC), and cholesterol (CHOL) were purchased from Sigma Aldrich (Oakville, ON, Canada). All chemicals, including potassium chloride (KCl), 1,1,1,3,3,3-hexafluoroisopropanol (HFIP), 4-(2-Hydroxyethyl) piperazine-1-ethanesulfonic acid (HEPES), decane, and butyl alcohol, were also purchased from Sigma Aldrich (Oakville, ON, Canada). Synthetic Aβ1-42 (HFIP) powder was obtained from rPeptide (Watkinsville, GA, USA). Gramicidin D powder was purchased from Sigma Aldrich (Oakville, ON, Canada).

### 2.2. Sample Preparation

#### 2.2.1. Lipid Preparation for Artificial Planar Bilayers

DPPC, POPC, and cholesterol were dissolved in a mass ratio 4:4:2 in a 9:1 (*v*/*v*) chloroform and methanol mixture to make 10 mg/mL lipid stock, which was stored at −20 °C. To prepare the lipid solution for BLM experiments, the lipid stock mixture was dried with nitrogen gas to completely evaporate chloroform and methanol. Then, dried lipids were resuspended in 50:1 decane and butyl alcohol at 10 mg/mL for BLM use [3].

#### 2.2.2. Amyloid β Oligomer Preparation

1 mg of synthetic Aβ1-42 powder was dissolved with 1 mL of HFIP and distributed into aliquots. After the evaporation of HFIP in the fume hood overnight for 24 h, dried Aβ1-42 samples were stored in the freezer at −20 °C. To produce solutions of small Aβ1-42 oligomers, Aβ1-42 was resuspended in a salt buffer (150 mM KCl and 20 mM HEPES) at a concentration of 122 μΜ. Aβ1-42 solution was incubated at 4 °C overnight (18 to 24 h) to obtain small Aβ1-42 oligomers and then used for extracellular electrophysiological measurement, according to the well-established protocol by Stine et al. [30].

#### 2.2.3. Gramicidin Preparation

A total of 10 µg of Gramicidin D powder was dissolved in 5.32 mL of ethanol to produce a 10 µM solution. The solution was then stored in a freezer at −20 °C and used for electrophysiological recordings without further modifications.

### 2.3. Black Lipid Membrane

The black lipid membrane (BLM) technique is an electrophysiological technique in which membrane permeability can be estimated by measuring the ion currents in a suspended lipid membrane [31]. It is widely used for studying the properties of protein–membrane interactions, as a measure of membrane damage induced by the protein binding and formation of ion channels and pores across the membrane [32]. The lipids form a suspended bilayer that can be used to study protein–membrane interactions by measuring different characteristics, including membrane stability, channel selectivity, permeability, and gating behavior [33,34].

#### Suspended Membrane Preparation

Membrane electrophysiological recording was conducted with an Orbit Mini instrument from Nanion Technologies (Munich, Germany) in the 150 mM KCl and 20 mM HEPES buffer. Artificial lipid membranes were suspended over 150 μm holes in MECA chips (Nanion Technologies, Munich, Germany) with four recording channels by painting via a Teflon sheet or bubbling via a 2 μL pipette. Aβ1-42 solution and gramicidin D were added directly into the chip, respectively. The final concentration of Aβ1-42 solution was 32 μM, and the final concentration of gramicidin D was 668 pM in the chip. The current increase induced by protein–membrane interaction was recorded at 100 mV with a 5 kHz sampling rate and SR/20 final bandwidth. The time duration, mean, and maximum value (*I*) of single current fluctuation caused by Aβ–membrane interactions were collected from the beginning of the current increase to the current drop for statistical analysis. At least 10 independent lipid membranes were produced for data collection.

### 2.4. Atomic Force Microscopy (AFM)

The existence and size of amyloid oligomers and fibrils was verified by AFM imaging. AFMs, configured in tapping mode (JPK NanoWizard, Bruker, Berlin, Germany), were used to image the Aβ1-42 oligomers and estimate their sizes. Dried protein samples were imaged in intermittent mode (air) with PPP-NCH probes (Systems for Research (SFR), Etobicoke, ON, Canada) with frequencies ranging from 297 kHz to 300 kHz and a spring constant of the probes of 42 N/m.

#### Amyloid Oligomers and Fibrils for AFM Imaging

The Aβ oligomers were prepared by suspending Aβ1-42 (HFIP) powder in a salt buffer (150 mM KCl and 20 mM HEPES) at 122 μM and incubating at 4 °C overnight. The amyloid fibrils were prepared with the same solution and incubated at room temperature overnight. Incubated Aβ1-42 was diluted with DI water, and the 10 μL protein solution was deposited on mica. After a 5-min waiting time, the Aβ-deposited mica was washed with 20 μL DI water four times and gently dried with nitrogen gas. Finally, samples were stored in a desiccator overnight before AFM imaging.

### 2.5. Data Collection and Statistical Analysis

For BLM data, the membrane conductance (*C*) is calculated as the ratio of current (*I*) to corresponding voltages (*V*) measured by BLM (C=I/V). Statistical analysis (ANOVA, Tukey’s test) was conducted with OriginPro 2020.

The AFM images were processed by Gwydion 2.56. Statistics and histograms for the z-height distribution of the Aβ1-42 were produced by MATLAB 2024. At least 3 samples were prepared, and at least 3 images were taken from each sample. More than 100 measurements were used for statistical analysis.

## 3. Results and Discussion

### 3.1. AFM Images of Aβ1-42 Oligomers and Fibrils

**Figure 2:**
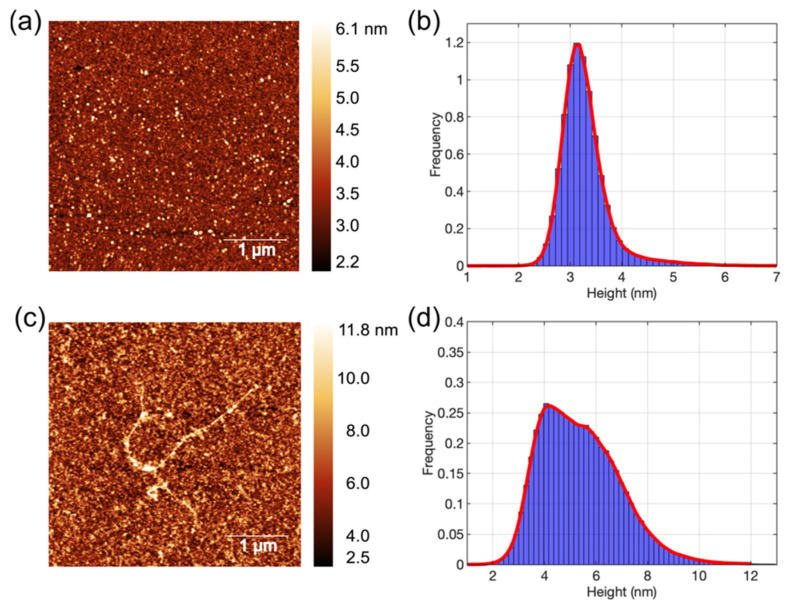
AFM images of Aβ1-42 aggregates and corresponding height distribution. (**a**) Aβ1-42 oligomer particles (white dots in the images), were incubated at 4 °C overnight, deposited on mica and imaged in air; and (**b**) the corresponding height distribution of oligomers that shows the z-height distribution. (**c**) Aβ1-42 fibrils incubated at room temperature overnight, and (**d**) the height distribution of fibrils. Size: 4 µm by 4 µm.

Aβ oligomers incubated at 4 °C and fibrils incubated at room temperature (~22 °C), which were deposited on mica substrate, were imaged by AFM, as shown in Figure 2.

AFM image (Figure 2a) confirmed that the Aβ prepared via Stine’s protocols [30] (i.e., via overnight incubation at 4 °C) is present as small oligomers. These Aβ1-42 appeared spherical in shape and mainly lie between 2.6 and 4 nm, according to the histogram of z-height distribution in Figure 2b. The average height of Aβ1-42 oligomers is 3.28 ± 0.49 nm (mean ± SD). AFM results identified that the majority of Aβ particles used for BLM and prepared according to our method are small oligomers with a diameter smaller than 4 nm. The oligomeric form of Aβ1-42 is known to have strong membrane affinity and toxicity, which can induce membrane permeation by forming ion channels or pores [6,7,10,20]. In comparison, Aβ1-42 peptides were also incubated at ambient temperature overnight and then imaged by AFM, as shown in Figure 2c. The AFM image showed the Aβ1-42 fibrils formed on the mica surface. Other than fibrils, larger Aβ1-42 oligomers were also seen. The histogram in Figure 2d displays the z-height distribution of Aβ1-42 aggregates, ranging from 3 to 10 nm and with an average z-height of 5.39 ± 1.54 nm. The Aβ1-42 aggregates incubated at room temperature were larger than those incubated at 4 °C, confirming that the aggregation of Aβ proteins used in this study at ambient conditions promotes further Aβ aggregation and fibrilization, as expected [30].

### 3.2. BLM Electrophysiology for Aβ1-42 and Gramicidin–Membrane Interaction

**Figure 3:**
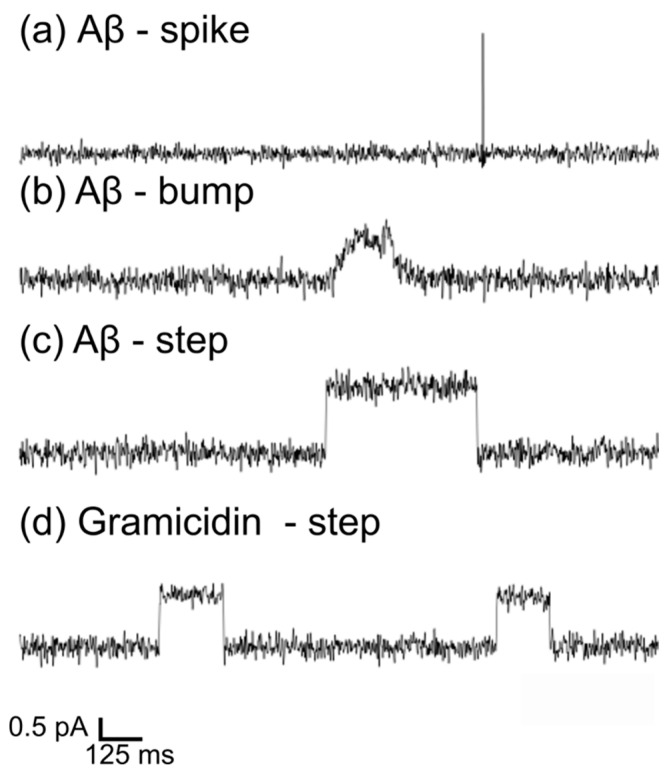
Ion current increases induced by Aβ–membrane interactions and gramicidin ion channel formation measured at 100 mV. (**a**–**c**) Aβ1-42-induced ion current flow with the shapes of spike, bump, and step. (**d**) Step-like currents due to gramicidin ion channel formation. Horizontal scale bar: 125 ms; vertical scale bar: 0.5 pA.

**Figure 4:**
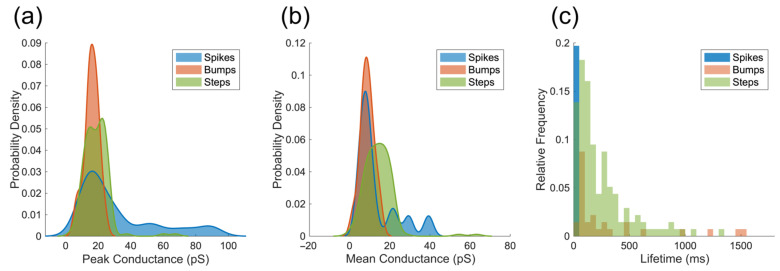
Conductance and lifetime (duration of the ion current) of ion current events in the membranes caused by oligomeric Aβ1-42: (**a**) peak and (**b**) mean conductance of three ion current events (spike, bump, step), (**c**) Lifetime of three events. Table 1 shows the value of conductance and lifetime.

**Figure 5:**
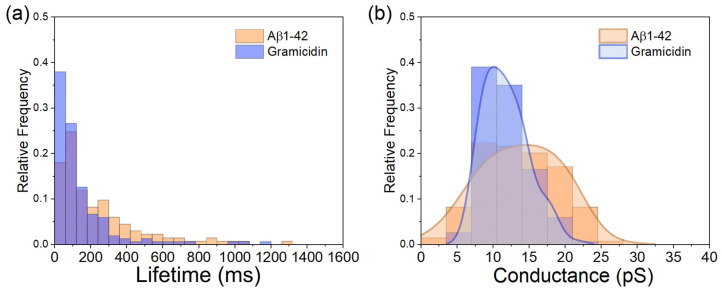
The comparison of Aβ ion channels and gramicidin channels. (**a**) Lifetimes of each channel and (**b**) ion channel conductance corresponding to step-like currents due to Aβ1-42 and gramicidin ion channels.

The Aβ1-42 oligomers produced by Stine’s protocols [30], as shown in Figure 2a, were applied to study Aβ–membrane interactions with BLM electrophysiology. BLM was used to measure the current change across a membrane incubated with small Aβ1-42 oligomers at 100 mV. Figure 3 displays increased current flux as the protein interacts with the suspended artificial membranes. The increased currents induced by Aβ1-42 could be categorized into three types: spike, bump, and step (Figure 3a–c), confirming multiple Aβ activities in lipid membranes. To refine our understanding of the amyloid-induced pores, we used the well-characterized gramicidin channel as a comparison. Combining the two gramicidin hemichannels in the lipid membrane generates a single transmembrane ion channel, as shown in Figure 1c. The ion current, due to the formation of a single gramicidin ion channel in the BLM, is featured in a step shape in Figure 3d. The statistical analysis of ion current events caused by protein–membrane interactions in planar lipid bilayers is displayed in Figure 4 and Figure 5.

### 3.3. Interactions of Aβ1-42 Oligomers with Suspended Lipid Membranes

The lifetime and conductance were measured to understand the characteristics of each type of event, as shown in Figure 4. The mean and maximum conductance of ion current events reflected the average and maximum membrane permeability due to the pore or ion channel formation.

The spike signal is characterized by an acute increase in current (Figure 3a) and an extremely short dwell time of tens of milliseconds (Figure 4c). The peak conductance of spike-like currents was significantly larger than step-like and bump-like currents (ANOVA, *p* < 0.05), implying that the momentary ion current leakage in the membrane during the spike event is more severe than in step and bump events. The short lifetime of these pores suggests that the membrane recovers and closes the pores instantaneously; therefore, the ion current flow disappears very quickly. These spike signals could be attributed to the instantaneous ion leakage due to the quick penetration of Aβ peptides in the lipid membrane, similar to the spike signals recorded for antimicrobial peptides [35]. In order to better understand the molecular activity behind the spike-like currents, the monomeric Aβ1-42 solution was applied to the lipid membranes, and the current fluctuation was also recorded and analyzed (see Supplementary Material S1). The preparation of Aβ monomers can also be found in the S1. Figure S2 in the S1 shows that the Aβ monomers can also induce spike-like ion currents, while their occurrence is low, suggesting less interaction between Aβ monomers and model lipid membranes. Despite the Aβ1-42 applied to BLM being predominantly oligomeric, there were still minor monomeric Aβ1-42 present in the samples, which could insert into lipid membranes [2,36], thereby causing spike-like currents in BLM output. Therefore, both monomers and small oligomers may induce minor dynamic instabilities in the membrane without forming a larger ion-conducting pore, thus contributing to spike formation.

In contrast to spikes, bumps exhibited a gradual increase and decrease in current with a longer lifetime (Figure 3b), which was rarely observed in past studies. This could be a random disruption or slow penetration by the Aβ1-42 oligomer. The step-like current (Figure 3c) manifests as a sudden increase in current, which is stable as a plateau for tens to hundreds of milliseconds before returning to the baseline (Figure 4c). The step signals appeared most frequently among the three types of signals (Table 1). Similar ion currents were observed in previous electrophysiological recordings in amyloid membrane studies [3,37] as well as with other types of proteins, such as alpha-hemolysin, alamethicin, and gramicidin, suggesting the formation of protein ion channels [38,39,40]. This indicates that the step-like shape induced by Aβ1-42 corresponds to the ‘open’ state of protein ion channels [35].

Another noticeable factor is the peak and mean conductance distribution of the three events, as shown in Figure 4a,b. Spike events have a broader distribution in conductance (both mean and peak), implying the randomness and uncertainty of the events. The conductance of bump and step events shows a narrower distribution, with the majority of the conductance (mean and peak) below 30 pS, except in a few cases that exceed 50 pS. This suggests more defined pore formation and correlates with the relatively constrained size range of Aβ1-42 oligomer clusters, as observed in this work.

Overall, three characteristic current shapes (step, spike, bump in Figure 3a–c and Figures S3–S5 in S1) induced by Aβ1-42 oligomers confirm that Aβ1-42 could interact with membranes in multiple ways [5]. Particularly, small Aβ1-42 oligomers prefer to form ion channels compared to the other two types of interaction. Our findings correlate with previous studies, which indicated that the oligomeric Aβ1-42 can form non-selective and voltage-independent ion channels, which allow the transport of Ca^2+^ and monovalent ions [3,7,20], regardless of the diverse sizes of Aβ1-42 oligomers.

### 3.4. Comparison of Aβ1-42-Induced Step-like Current with Gramicidin

The step-like currents appeared in Aβ– and gramicidin–membrane interaction, implying the formation of Aβ and gramicidin ion channels. The lifetime and the mean conductance of the two ion channels are compared in Figure 5. A 75% proportion of gramicidin ion channels have a lifetime smaller than 165 ms. In comparison, close to 50% of Aβ1-42 ion channels remain in the lipid membrane for longer than 200 ms (Figure 5a). The lifetime and formation probability of gramicidin channels depend on the duration of subunit pairing, which is influenced by multiple factors, such as the surface density of gramicidin subunits, the composition of lipid membranes, membrane elasticity and the properties of the membrane–aqueous interface [40,41]. Channel dissociation, caused by the separation of subunits, increases the uncertainty and leads to a shortened gramicidin channel lifetime. In contrast, Aβ ion channels, which are formed by Aβ oligomers, do not rely on such subunit matching and may be composed of a different number of Aβ peptides as previously suggested [19,22]. Nevertheless, most of the Aβ ion channels (>75%) in our study still had a shorter lifetime (<400 ms) than the claim in previous studies so that, in summary, the typical lifetime for Aβ ion channels was above 500 ms [6].

The mean conductance of gramicidin channels was 11.73 ± 3.13 pS (*n* = 151), while the mean conductance of Aβ channels was 14.40 ± 7.64 pS (*n* = 137). Statistically, based on ion current response, the Aβ channels were significantly larger than the gramicidin channels (*t*-test, *p* < 0.05). The distribution of conductance values for the amyloid channels is more dispersed than that of the gramicidin channels, completely overlapping the range of gramicidin conductance values, as demonstrated by the conductance distribution plot (Figure 5b). This indicates that some small Aβ oligomers could form ion channels with conductance equivalent to those formed by gramicidin, while others could be larger. Assuming that both gramicidin and amyloid channels are cylindrical, the relationship between conductance and pore size can be expressed by Equation (1) [7,42],

(1) $$G=\frac{\sigma \pi d^{2}}{4l}$$

where *σ* is the conductivity of the solution, *d* is the inner diameter of the nanopore, and *l* is the height of the channel, which correlates to the length of the hydrophobic tails in lipid membranes [7,42]. The length of the gramicidin channel, composed of two subunits, is 26 Å [43]. The length of the small Aβ1-42 ion channel has been reported to be ~3 nm (equivalent to 30 Å) [7], which is aligned with the size of the oligomers we used, as determined with AFM. Since we used the same lipid model and ionic solutions, it is reasonable to assume that the σ were the same in the measurement of gramicidin and Aβ1-42 channel conductance. The diameter of the gramicidin A ion channels, which is the main component of gramicidin D, is approximately 4 Å in phospholipid membranes [44]. Based on the length ratio and the average conductance ratio of gramicidin (11.73 pS) to Aβ1-42 (14.40 pS) ion channels, the average diameter of the Aβ ion channels calculated by Equation (1) was 4.76 Å, which is slightly larger than gramicidin A channel but at the same magnitude level. In addition, the more spread conductance distribution of Aβ1-42 ion channels in Figure 5b elucidates that the diameters of Aβ1-42 channels have a broader range than that of the gramicidin A channel.

Previous studies suggested that the Aβ oligomers form ion channels with β-barrel structure in the membranes [7,16,20,45,46]; the typical conductance of these Aβ channels is larger than 250 pS [6]. Some studies also found Aβ-induced conductance larger than 200 pS in artificial lipid membranes [7] and neurons [47] via electrophysiological recordings. Our results for the Aβ ion channel (14.40 ± 7.64 pS) are more than 10 times smaller than the previous observations [6,7,47]. While we cannot exclude the influence of the differences of electrolytes and lipid composition between different experimental setups, these differences are most likely to have only a small effect on variances in conductance. On the other hand, the large difference in the conductance may suggest that Aβ ion channels can be variable in pore size and in the number of Aβ peptides forming the channel. The inner diameter of Aβ ion channels is reported to be 1 to 2 nm, with the size of Aβ oligomers ranging from 6 to 10 nm [48,49,50,51]. Bode et al. experimentally confirmed that the ion channels composed of Aβ1-42 oligomers with sizes of 5 to 20 nm are capable of forming ionic pores with a diameter ranging from 1.7 to 2.4 nm [20]. In our experiments, the small Aβ1-42 oligomers with a mean size of 3.28 ± 0.49 nm formed ion channels with a pore size below 5 Å, which have not been reported before.

Other than small Aβ1-42 ion channels generating small ion currents reported in this study, the large current flow above 20 pA with irregular shape represents the destructive effect of Aβ oligomers on membranes, which has been observed in previous amyloid studies [3,52]. This shows the direct Aβ damage to the membrane integrity, resulting in dramatic ion leakage. The diversity of the current flux, characterized by ion currents of various shapes and durations, can be caused by the multiple mechanisms of Aβ–membrane interaction, diverse Aβ sizes, and lipid membrane compositions [53].

The electrophysiological measurements from BLM reveal the various damage routes of Aβ1-42 to membranes, despite that thorough classification of the current features and corresponding activity is still ambiguous. The actual Aβ–membrane interaction associated with the bump-shaped current (Figure 3b) remains unclear. Previous studies of Aβ–membrane interactions using electrophysiological recordings have classified the current features as step-like, spike-like, and burst events [3,54,55]. In addition, according to the antimicrobial hypothesis [14,15,56], the Aβ–membrane interaction can be similar to the behavior of antimicrobial peptides (AMP), which can also insert into the membranes and cause pore formation [15,57]. Saigo et al. categorized these current features in electrophysiological recordings and proposed protein-membrane models to explain the interaction mechanisms for AMPs, including Clavanin A, Cecropin A, and magainin [35]. The classification includes steps, multilevel, erratic, and spike signals, which are also observed in our Aβ-membrane measurements, according to the shapes of the currents [35]. In addition to the ion channel mechanism, Aβ can harm lipid membranes through a detergent-like effect, carpet-like mechanism, oxidation, or interaction with transmembrane protein receptors [6]. These various interacting pathways can affect membrane integrity both in the short and long term.

Our study revealed that small Aβ1-42 oligomers at the early aggregation stage can form small ion channels in the lipid membrane with an ionic signature comparable to gramicidin channels, among other features observed, such as spikes and bumps. Despite large amyloid aggregates being thought to bring more destruction to membrane stability by inducing huge conductance fluctuation according to planar lipid bilayers studies, cell viability studies showed the opposite size-dependency on amyloid toxicity—smaller Aβ oligomers (1 to 2 nm) showed more cytotoxicity than larger ones [21].

## 4. Conclusions

In this work, we studied the interaction of small Aβ1-42 oligomers with an average size of ~3 nm (confirmed by AFM) with suspended planar lipid bilayers using black lipid membrane electrophysiology in comparison with antimicrobial gramicidin channels. The results of electrophysiological measurements across the suspended lipid membranes incubated with Aβ oligomers showed distinct current shapes, including step-like currents with short lifespans. The step-like current can be identified as the ionic transport due to an ion channel formed by small Aβ1-42 oligomers in the membrane. This ion current signature of Aβ1-42 ion channels resembles that of gramicidin D channels. These results identify the new smaller Aβ1-42 iοn channels and provide a deeper understanding of the molecular mechanism of Aβ-induced membrane disruption.

## Figures and Tables

**Table 1:** Conductance and lifetime of oligomeric Aβ-induced current signals. The data are shown as mean ± SD. The number of membranes (N) indicates the count of membranes where a specific type of signal was observed. Sample size (n) represents the total number of signals observed.

Signal Type	Number of Membranes (N)	Sample Size (n)	Peak Conductance (pS)	Mean Conductance (pS)	Lifetimes (ms)
Spikes from oligomeric Aβ solution	4	27	31.50 ± 24.34	13.38 ± 10.28	9.04 ± 4.58
Bumps from oligomeric Aβ solution	6	31	15.92 ± 4.34	8.79 ± 3.34	321.30 ± 426.47
Steps from oligomeric Aβ solution	7	137	18.85 ± 7.98	14.40 ± 7.64	277.20 ± 371.14

## Data Availability

The original contributions presented in this study are included in the article and Supplementary Materials. Further inquiries can be directed to the corresponding author.

## Supplementary Materials

The following supporting information can be downloaded at: https://www.mdpi.com/article/10.3390/membranes15070204/s1, Figure S1: Representative trace of lipid bilayers (without Aβ1-42) in response to voltage fluctuations from −100 to 100 mV. Figure S2: Representative trace of Aβ monomers interacting with the lipid membrane with ‘spike’ current caused by Aβ monomers. Figure S3: Step-like currents caused by oligomeric Aβ, suggesting the insertion of Aβ ion channels into the lipid membranes. Figure S4: Spike-like currents caused by the oligomeric Aβ solution. Figure S5: Two representative bump-like currents caused by oligomeric Aβ solution. Table S1: Conductance and lifetime of ion current signals caused by monomeric and oligomeric Aβ1-42 solutions.
