# Supplementary material for: Amyloid β 1-42 Can Form Ion Channels as Small as Gramicidin in Model Lipid Membranes
Source: Membranes (Basel). 2025 Jul 8;15(7):204. doi: 10.3390/membranes15070204 (PMC12299968; doi:10.3390/membranes15070204)
Supplement: Supplementary file 1 [file membranes-15-00204-s001.zip › membranes-3619402-supplementary.pdf]

Supplementary Material-S1

Amyloid β 1-42 can form Ion Channels as Small as Gramicidin in Model Lipid Membranes

Yue Xu [1], Irina Bukhteeva [1,2], Yurii Potsiluienko [1] and Zoya Leonenko [1,2,3,*]

[1] Department of Physics and Astronomy, University of Waterloo, Waterloo, ON N2L 3G1, Canada; yue.xu@uwaterloo.ca (Y.X.); ibukhteeva@uwaterloo.ca (I.B.); ypotsiluienko@uwaterloo.ca (Y.P.)
[2] Waterloo Institute for Nanotechnology, University of Waterloo, Waterloo, ON N2L 3G1, Canada
[3] Department of Biology, University of Waterloo, Waterloo, ON N2L 3G1, Canada
* Correspondence: zleonenk@uwaterloo.ca

1.1 Blank control of DPPC/POPC/CHOL membrane (no Aβ)

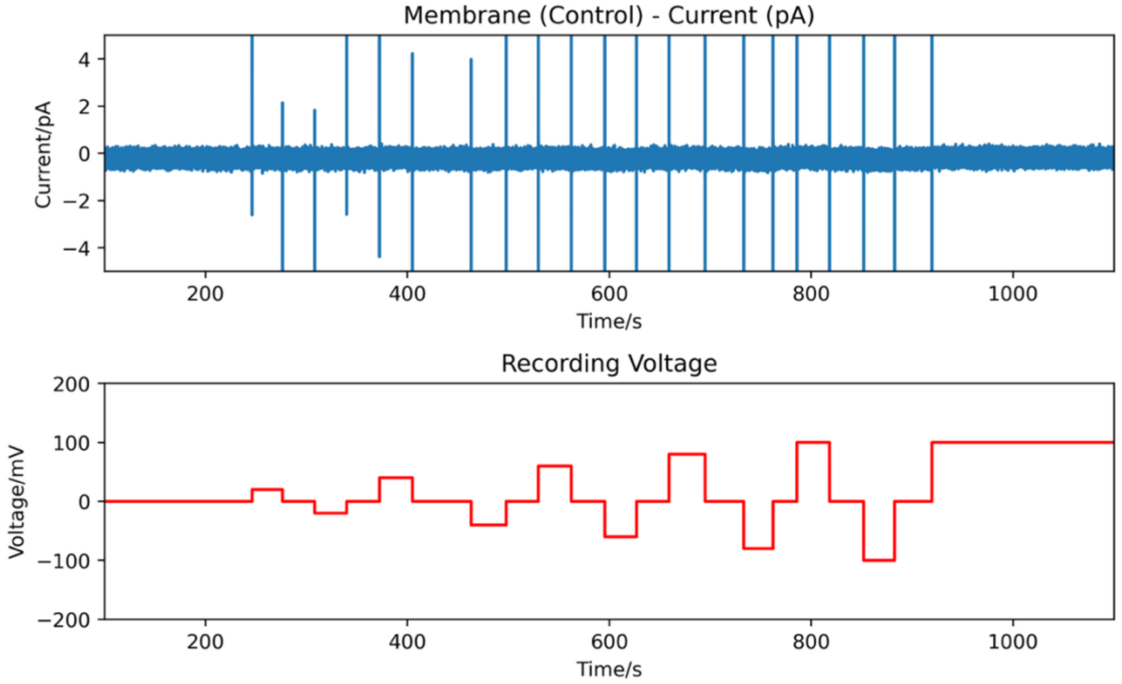

Figure S1. Representative trace of lipid bilayers (without Aβ1-42) in response to voltage fluctuations from -100 to 100 mV. Voltage step size: 20 mV.

During electrophysiological recordings, our lipid bilayers remain stable in response to voltage fluctuations ranging from -100 to 100 mV (Figure 1). However, spike-like currents only emerge at the transition points of voltage changes. The ion current for a single lipid bilayer without Aβ or

gramicidin stays near zero at a constant voltage.

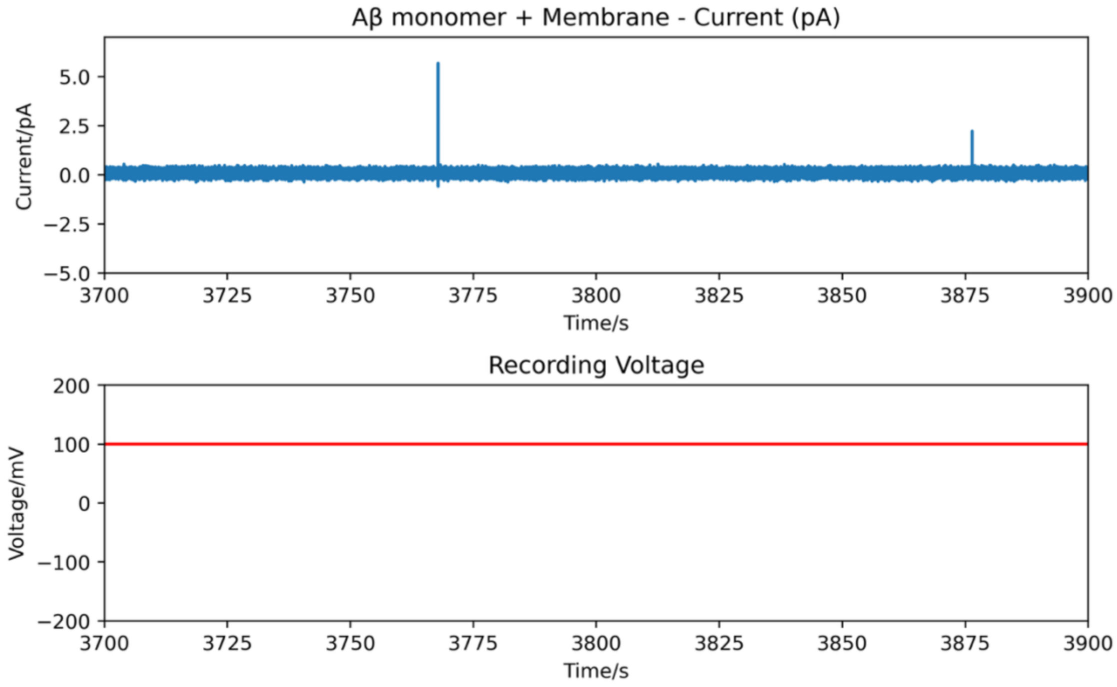

Figure S2. Representative trace of Aβ monomers interacting with the lipid membrane with 'spike' current caused by Aβ monomers.

1.2 Monomeric Aβ experiments

Preparation of Aβ monomers

The Aβ monomers were produced by direct resuspension of dried Aβ (HFIP) aliquots in a salt buffer (150 mM KCl and 20 mM HEPES) at a concentration of 122 µM without overnight incubation. They were used in BLM electrophysiological measurement immediately after resuspension.

Spike-like currents caused by Aβ monomer solutions

While the Aβ monomers are less likely to bind to lipid membranes, occasionally, they induce spike-like ion currents when interacting with membranes, as shown in Figure 2. The lifetime and conductance of monomeric Aβ-induced ion current signals are presented in Table 1, compared with the characteristics of oligomeric Aβ-induced signals.

Table S1. Conductance and lifetime of ion current signals caused by monomeric and oligomeric Aβ1-42 solutions.

**Signal Type**	**Number of Membranes (n)**	**Sample size (N)**	**Peak Conductance (pS)**	**Mean Conductance (pS)**	**Lifetimes (ms)**
**Spikes from monomeric Aβ solution**	9	17	26.53 ± 18.39	10.68 ± 7.35	6.20 ± 2.21
**Spikes from oligomeric Aβ solution**	4	27	31.50 ± 24.34	13.38 ± 10.28	9.04 ± 4.58
**Bump from oligomeric Aβ solution**	6	31	15.92 ± 4.34	8.79 ± 3.34	321.30 ± 426.47
**Step from oligomeric Aβ solution**	7	137	18.85 ± 7.98	14.40 ± 7.64	277.20 ± 371.14

1.3 Other representative traces of signals caused by Aβ oligomer solution

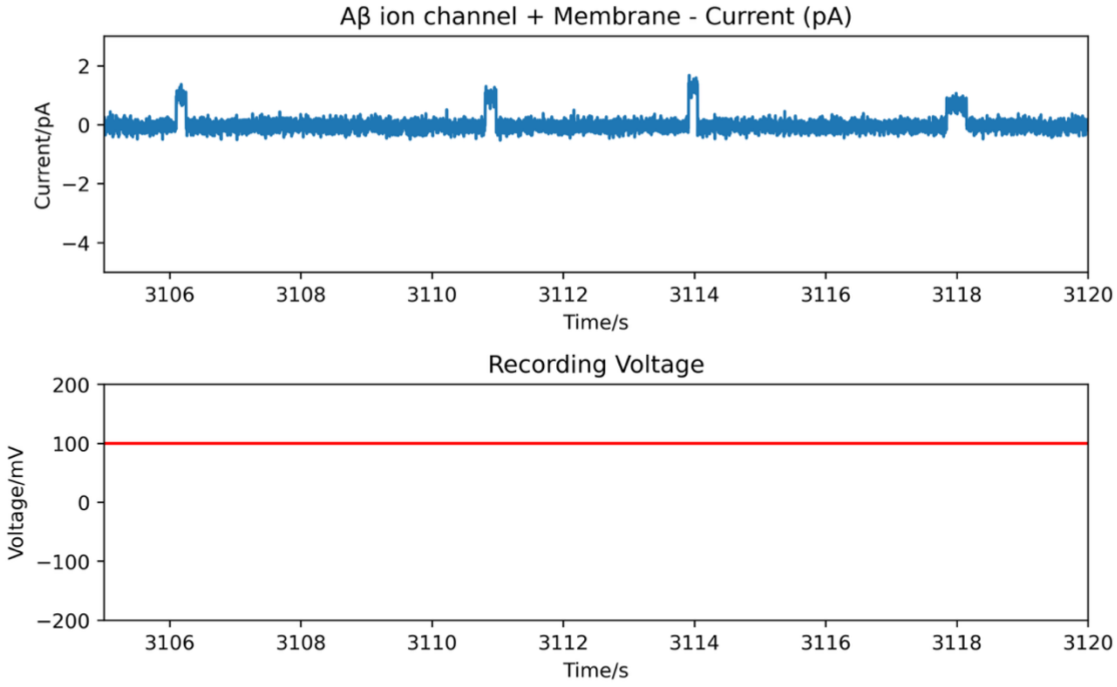

Figure S3. Step-like currents caused by oligomeric Aβ, suggesting the insertion of Aβ ion channels into the lipid membranes.

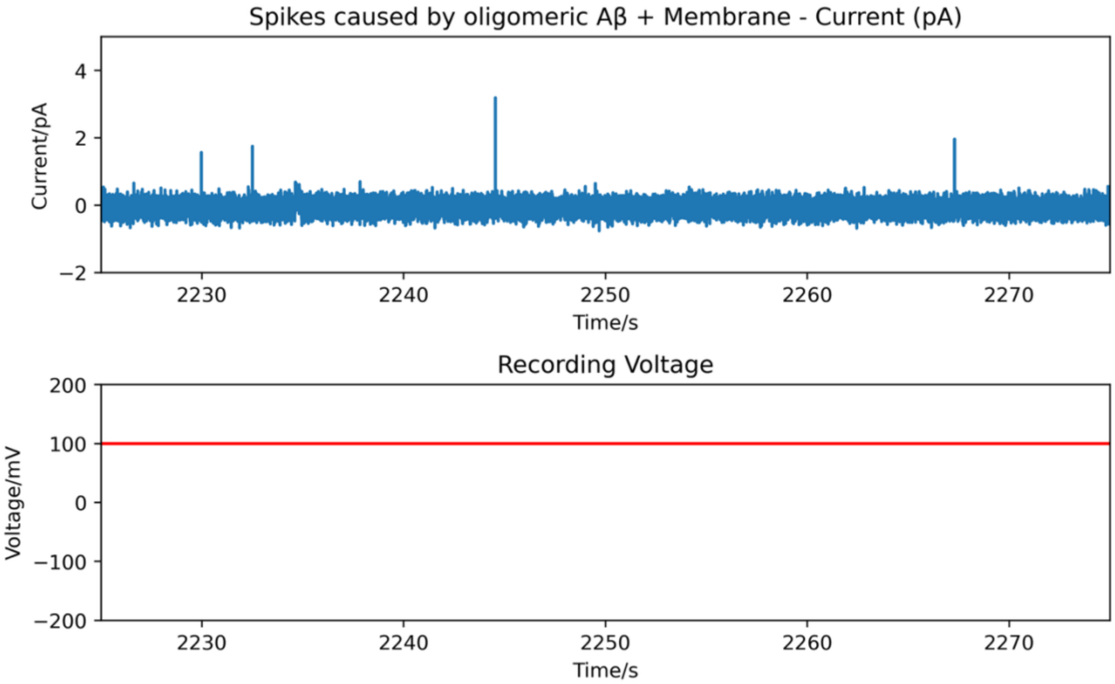

Figure S4. Spike-like currents caused by the oligomeric Aβ solution.

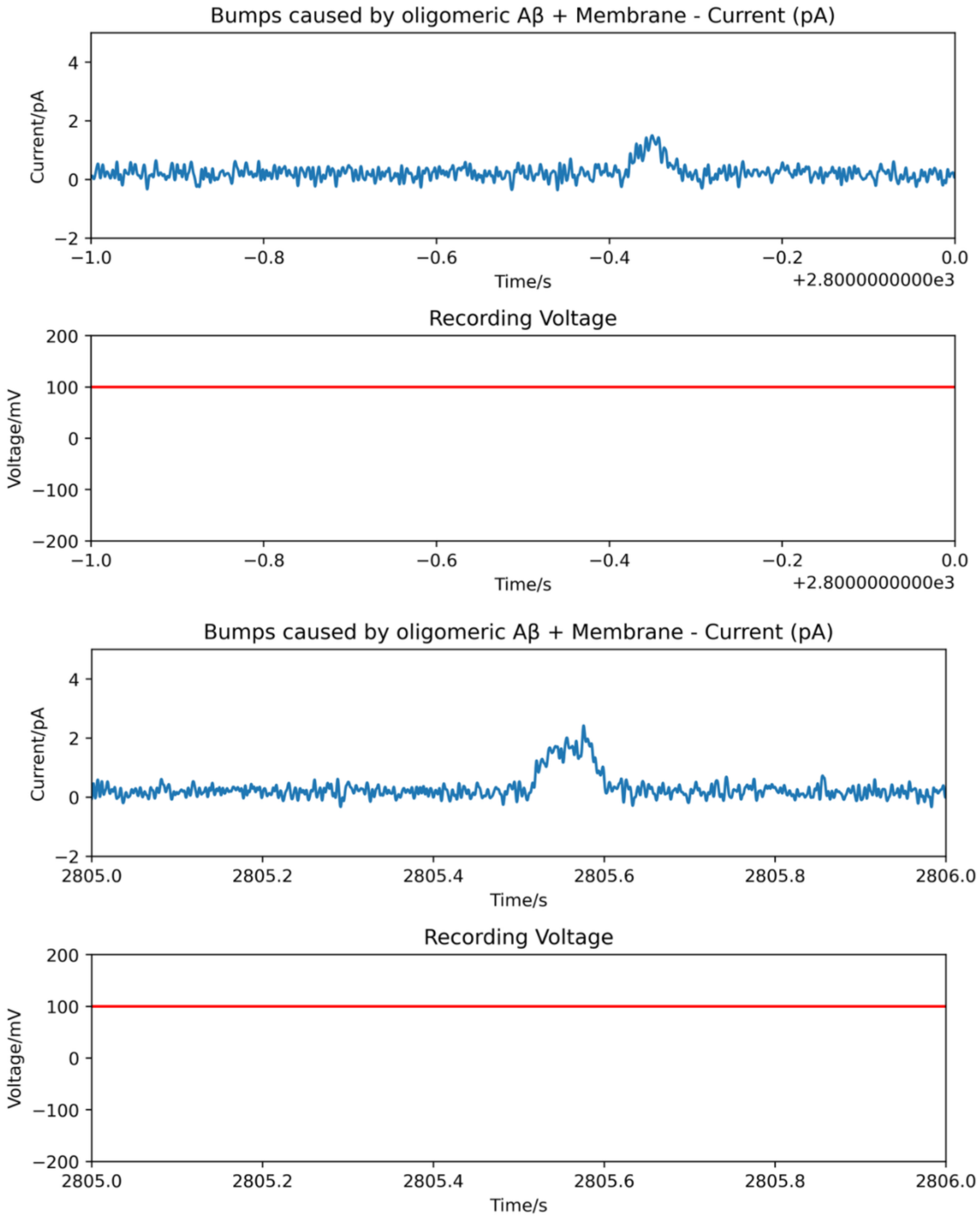

Figure S5. Two representative bump-like currents caused by oligomeric Aβ solution.
